# Outcomes and Impact of Training and Development in Health Management and Leadership in Relation to Competence in Role: A Mixed-Methods Systematic Review Protocol

**DOI:** 10.15171/ijhpm.2016.138

**Published:** 2016-10-17

**Authors:** Reuben Olugbenga Ayeleke, Nicola North, Katharine Ann Wallis, Zhanming Liang, Annette Dunham

**Affiliations:** ^1^Health Systems Section, School of Population Health, University of Auckland, Auckland, New Zealand.; ^2^Department of General Practice and Primary Health Care, School of Population Health, University of Auckland, Auckland, New Zealand.; ^3^Department of Public Health, School of Psychology and Public Health, La Trobe University, Melbourne, Australia.

**Keywords:** Health Management, Clinical Leadership, Competence, Training, Professional Development Programmes

## Abstract

**Background:** The need for competence training and development in health management and leadership workforces has been emphasised. However, evidence of the outcomes and impact of such training and development has not been systematically assessed. The aim of this review is to synthesise the available evidence of the outcomes and impact of training and development in relation to the competence of health management and leadership workforces. This is with a view to enhancing the development of evidence-informed programmes to improve competence.

**Methods and Analysis:** A systematic review will be undertaken using a mixed-methods research synthesis to identify, assess and synthesise relevant empirical studies. We will search relevant electronic databases and other sources for eligible studies. The eligibility of studies for inclusion will be assessed independently by two review authors. Similarly, the methodological quality of the included studies will be assessed independently by two review authors using appropriate validated instruments. Data from qualitative studies will be synthesised using thematic analysis. For quantitative studies, appropriate effect size estimate will be calculated for each of the interventions. Where studies are sufficiently similar, their findings will be combined in meta-analyses or meta-syntheses. Findings from quantitative syntheses will be converted into textual descriptions (qualitative themes) using Bayesian method. Textual descriptions and results of the initial qualitative syntheses that are mutually compatible will be combined in mixed-methods syntheses.

**Discussion:** The outcome of data collection and analysis will lead, first, to a descriptive account of training and development programmes used to improve the competence of health management and leadership workforces and the acceptability of such programmes to participants. Secondly, the outcomes and impact of such programmes in relation to participants’ competence as well as individual and organisational performance will be identified. If possible, the relationship between health contexts and the interventions required to improve management and leadership competence will be examined

## Background

The healthcare system is complex, dynamic, constantly evolving, and a target of repeated reforms. These reforms have brought about changes to roles of those in health management and leadership and to the associated competence required to perform these roles.^1^

The importance of developing and improving the competence of health management and leadership workforces through training and development programmes have been emphasised.^2,3^ However, the effects of such programmes on competence and individual or organisational performance have not been assessed and synthesised systematically. Thus, questions arise as to whether or not the various interventions aimed at addressing the gaps in health management and leadership competence actually lead to improvement in individual and organisational performance.^4^

Health management and leadership workforces play a crucial role in effective healthcare delivery, and in maximising the gains of the various reforms in the sector.^3^ It is, therefore, pertinent to review available evidence of the overall effects of interventions to improve management and leadership competence. This, in turn, will inform appropriate policy formulation to enable competence improvement, and the development of evidence-informed and sustainable training and development programmes. With the recent upsurge in research in health management and leadership competence, this review is timely and necessary to ascertain where further research is required.

This review will make use of a mixed-methods research synthesis which could provide answers to a wide range of research questions which a single method approach might not address comprehensively.

## Aim and Objectives

This review aims to critically appraise and synthesise empirical evidence of the outcomes and impact of training and development programmes in relation to the competence of health management and leadership workforces.

## Review Questions

This review aims to answer the following questions:

1. What training and development programmes are used to improve the competence of health management and leadership workforces, and are acceptable to participants?
2. Do training and development programmes improve the competence of health management and leadership workforces?
3. What are the characteristics of training and development programmes that are effective and appropriate for improving the competence of health management and leadership workforces?

## Methods

This review will make use of the segregated approach to mixed-methods research synthesis.^5^ Thus, qualitative and quantitative studies as well as the respective components of primary level mixed-methods studies will be analysed into two separate sets of syntheses. Where appropriate, quantitative and qualitative data will then be integrated into a single synthesis using Bayesian method.^6^

The review will, therefore, involve:

- A synthesis of studies on the views, perspectives, or experiences of health management and leadership workforces on the outcomes and impact of the various training and development initiatives in relation to their competence and performance in roles;
- A synthesis of evidence of the effectiveness of training and development initiatives for improving the competence of health management and leadership workforces.

### Inclusion and Exclusion Criteria

#### Types of Study

- Only empirical studies with qualitative, quantitative, or mixed-methods designs will be eligible for inclusion. Studies reporting the findings of empirical research without giving details of the primary studies will be excluded. Reviews and commentaries will also not be eligible for inclusion;
- Qualitative research of any design which focused on the perspectives, experiences, or narratives of participants will be considered for inclusion. This will include designs such as general inductive design, grounded theory, action research, ethnography, among others;
- Quantitative design will include experimental, quasi-experimental and non-experimental, or epidemiological study designs;
- Mixed-methods design will be studies that employed mixed primary level qualitative and quantitative research approaches;
- Only studies published in English language from 2000 to date will be eligible for inclusion as we consider the most recent publications to be more relevant to the current research efforts. However, seminal or germinal studies published prior to 2000 will be considered for inclusion.

#### Contexts or Settings

This review will examine the managerial and leadership competence of health managers and clinical leaders of health services and health organisations from different countries in relation to the health context in which the identified competence is required. Health context will be considered from the angle of the types of work and setting, which could include any of the following organisational settings:

- Public sector;
- Private sector;
- Non-governmental organisations, charitable or voluntary organisations.

Studies that selected participants from the following work settings will be included in this review:

- Hospital, including secondary and tertiary level care;
- Primary healthcare services;
- Community health services;
- Residential care services;
- Government ministries, departments and agencies responsible for policy formulation, funding, regulation, and administration;
- Other work settings considered by the reviewers to be health sector or related to health sector.

#### Participants

- Participants of the primary studies will be individuals with existing roles as managers or leaders in the health sector;
- Generalist managers, professional health service managers, and individuals trained in other professions (eg, clinicians, IT personnel) but were involved in management and/or leadership development activities by virtue of their roles or positions as managers and/or leaders (eg, project managers, team leaders) will be eligible for inclusion;
- All levels of management or leadership positions will be included: frontline, middle, senior, and executive levels;
- Studies examining the competence of health professionals who perform dual roles eg, clinical and management/leadership roles, will be eligible for inclusion, providing separate data are available for management and/or leadership roles;
- Studies investigating the competence of both management and non-management staff will be included, providing separate data are provided for participants in management and/or leadership roles;
- Studies examining the competence of prospective or newly employed health managers and/or leaders with no prior management and/or leadership experience will be excluded; studies investigating the competence of individuals previously engaged in management and/or leadership positions will also be excluded.

#### Interventions or Exposures

- This review will consider ‘interventions’ as any specific initiatives (both formal and informal) intended to improve the competence of personnel responsible for management and leadership roles in healthcare organisations;
- Such initiatives may include coaching, mentoring, supervision, continuing professional development, training, formal education, contextualised learning, or other specific initiatives which are considered by this review to be appropriate for improving the competence of health managers and leaders;
- Studies on measures or incentives to improve the performance of health workers, including management and leadership workforces, but which did not address management or leadership competence, will be excluded.

#### Comparators or Control

Where applicable, comparators or control will be non-exposure to the intervention of interest or exposure to an intervention which is considered by this review not to be related to improving the competence of health management and leadership workforces.

#### Outcome and Impact Measures

In this review, the terms ‘outcomes and impact’ will be defined, using the operational definitions of the Centre for Non-profit Management (CNM), the United States^7^ as guides. Thus, outcomes refer to the measurable and specific effects of interventions (in the short and medium terms) such as the number of participants demonstrating changes in competence at the end of interventions. Impact, on the other hand, refer to the broad and long-term effects of interventions, either negative or positive, intended or un-intended; for example, changes in individual and organisational performance following completion of interventions.

Outcome and impact measures that will be considered in this review are those that will assist in addressing the research questions. Studies will not be excluded solely on the basis of not reporting any relevant outcome or impact measures, providing their designs, settings, participants and interventions meet the eligibility criteria for inclusion. Authors of such studies will be contacted for information on outcome and/or impact measures that could be relevant to the review. Final assessment of the outcomes and impact of training and development programmes will be based on studies that reported those data and on studies whose authors provided relevant information on outcome and/or impact measures following contact.

For the purpose of data synthesis and because of the possibility of studies reporting their findings at different end points, studies assessing the effects of interventions within each of the following time intervals will be regarded as similar in relation to timing of effect measures (such studies may be combined in meta-analysis or meta-synthesis, providing they are similar with respect to other characteristics ie, settings, participants, interventions and outcome or impact measures):

1. Immediately at the end of intervention and up to three months after completion of intervention (short-term measures);
2. More than three months and up to six months after completion of interventions (medium-term measures);
3. More than six months and up to 12 months after completion of interventions (long-term measures).

The following primary and secondary outcome and impact measures will be considered:

#### Primary Outcome and Impact Measures

These will be assessed as the direct effects of interventions related to participants’ managerial and/or leadership competence and performance in roles.

These will be assessed as the direct effects of interventions related to participants’ managerial and/or leadership competence and performance in roles.

Primary *outcome* measures will consist of the following:

- Objective assessment of competence or proficiency by participants, using standardised instruments;
- Objective assessment of competence or proficiency by stakeholders eg, participants’ superior officers and professional colleagues, using validated instruments;
- Subjective assessment of competence or proficiency from participants’ perspectives;
- Subjective assessment of competence or proficiency from stakeholders’ perspectives eg, participants’ superior officers and professional colleagues.

Primary *impact* measures will be:

- Participants’ performance in the related area following specific training or professional development inputs eg, financial performance, managerial and/or leadership performance.

#### Secondary Outcome and Impact Measures

These will be considered as the indirect effects of interventions related to individuals (participants) and/or organisations.

Secondary *outcome* measures will consist of:

- Costs associated with the intervention;
- Participants’ evaluation of the intervention in terms of their level of satisfaction with and acceptability of the interventions.

Secondary *impact* measures will consist of:

- Organisational performance in the related area following specific training or professional development inputs eg, quality of service, customer satisfaction, change in infection rates, morbidity and mortality rates, staff turnover.

### Search Strategy

A comprehensive search for relevant studies will be undertaken. The main concepts addressed in the review (ie, impact, health leadership or management, competence, training or development and performance or outcome) will be searched using strings of subject headings (from either the controlled vocabulary or thesaurus as appropriate) and free-text. The search strategy will be designed by combining search strings for each of the key concepts so as to identify studies that focus on, for example, health AND (management or leadership) AND (training or development) AND competence. A number of search strategies will be combined and utilised, depending on the database, to locate studies,

Only studies published in English language from 2000 to date will be included in this review. However, seminal studies published prior to 2000 will be considered for inclusion. Subject-specific electronic databases will be searched to identify studies relevant to the review. Other electronic sources will be databases dedicated to review of research, dissertations and theses. For the full list of electronic databases and catalogue to be searched, see Appendix 1.

Other potential sources will be searched to identify relevant studies. These will include hand searching of reference lists of included studies, conference proceedings, grey literature and websites of relevant health professional bodies, World Health Organization (WHO), and appropriate government agencies.

### Data Extraction and Analysis

#### Selection of Studies

Search results will be uploaded into RefWorks or EndNote so as to identify and remove any duplicates. The list of titles and abstracts generated by the search will be screened independently by two reviewers to identify potentially relevant articles. Full-text articles of potentially relevant articles will be retrieved and assessed independently for eligibility by two reviewers in accordance with the inclusion and exclusion criteria described above. Any difference in opinion between the two reviewers will be resolved through discussion or consultation with a third reviewer.

#### Data Extraction and Management

Two reviewers will extract data independently from the included studies using data extraction forms specifically developed for each study design. Disagreement, if any, between the reviewers will be resolved through discussion or by seeking the opinion of a third reviewer. Data will be extracted from each of the included studies with respect to study designs, settings, participants, interventions, and outcome and impact measures considered relevant to the review questions. For qualitative studies, key themes will be coded according to the content of the findings of each study, using NVIVO software. For quantitative research, descriptive and outcome data will be checked and double entered into RevMan data management software. Where data are missing, unclear or presented in a form that cannot easily be extracted, study authors will be contacted for clarification or assistance with the process of data extraction.

#### Assessment of Methodological Quality/Risk of Bias of Studies

This review will make use of methodologically appropriate tools to critically appraise the methodological quality of included studies. These are the Effective Public Health Practice Project (EPHPP) tool for quantitative studies,^8^ the Critical Appraisal Skill Programme (CASP) tool for qualitative studies,^9^ and a combination of these tools for mixed-methods designs.

The methodological quality of quantitative studies (ie, the risk of bias) will be assessed with respect to appropriateness of study design, process used in selecting participants, control for confounding factors, blinding of participants and/or personnel, including outcome assessors (researcher’s role and influence), follow up length, withdrawals/losses to follow up and reasons for withdrawals, data collection and analysis methods.

Qualitative studies will be assessed in relation to the appropriateness of qualitative design for the research question, appropriateness of the recruitment process, adequate description of data collection process and data analysis (rigor), researcher’s role and influence (bias) as well as the process to ensure credibility of the findings through, for example, triangulation and respondent validation.

Mixed-methods studies will be separated into their respective quantitative and qualitative components and assessed using the respective assessment tool for each study design. The outcomes of the assessment of the two separate components will be incorporated into the respective findings of the methodological assessment of the primary level quantitative and qualitative studies.

The overall quality of each study will be graded as ‘very good’ (high quality), ‘moderate’ or ‘weak’ (low quality) based on the average score for the domains assessed. No studies will be excluded on the basis of being of low quality; rather, the effect of inclusion of such low quality studies will be explored by using sensitivity analysis where appropriate. This will be done by considering the effect of removal of studies rated as low quality on the findings of both quantitative and qualitative studies.

Two reviewers will assess independently the methodological quality of studies. Any disagreement will be resolved through discussion or by consulting with a third reviewer.

#### Data Synthesis

##### Quantitative Studies

The effectiveness or otherwise of each intervention in improving the competence of participants will be determined by calculating, where appropriate, the effect size estimate for each intervention. Effect sizes will be expressed as odds ratio (OR) or risk ratio (RR) for categorical data and weighted mean difference (MD) for continuous data and 95% CIs will be calculated.

Studies that are sufficiently similar with respect to settings, participants, interventions and outcome or impact measures will be combined in meta-analysis. Where it is not possible to conduct meta-analysis due to substantial heterogeneity among studies, findings from individual studies will be presented in narrative analysis, using tables and figures as appropriate.

##### Qualitative Studies

Data from studies that examined the views, perspectives or experiences of participants will be synthesised thematically by two reviewers in accordance with the existing methods for thematic synthesis of qualitative research.^10^ Findings of the included studies will be assembled and rated according to their quality and then categorised into themes and sub-themes based on their similarity in meaning. The emerging themes and sub-themes will be examined to see how they are related to the research questions.

Where studies are sufficiently similar with respect to settings, participants, interventions and outcome or impact measures, a single set of synthesised findings will be produced by pooling the emerging themes in a meta-synthesis. Otherwise, the findings from each study will be presented in narrative form in an evidence table, taking into consideration the quality and consistency of the findings as well as their applicability to the research questions.

##### Mixed-Methods Studies

Findings of mixed-methods studies will be separated into their respective quantitative and qualitative components. Where possible, the quantitative component will be included in the quantitative synthesis while the qualitative component will be incorporated into the qualitative synthesis.

##### Aggregation of Data/Mixed-Methods Synthesis

Findings from quantitative and qualitative studies will be analysed separately (as previously described) using the segregated approach^5^ and integrated into two separate sets of data. Results from the initial quantitative syntheses will then be converted into textual descriptions (qualitative themes) using Bayesian method as described by Crandell and colleagues.^6^ Textual descriptions and the initial synthesised findings from qualitative studies that are mutually compatible or sufficiently similar will then be combined to generate mixed-methods syntheses using appropriate mixed-methods research analytical/assessment instrument. However, where it is not possible to conduct meta-analysis or meta-synthesis due to insufficient studies or substantial heterogeneity (differences) among studies, findings from individual quantitative studies will be converted into textual forms and then combine with compatible themes from individual or synthesised qualitative findings.

#### Subgroup Analysis

Subject to the availability of sufficient studies, subgroup analysis will be conducted to examine the influence of health system contexts on training and development needs of health management and leadership workforces. Studies will be subgrouped by:

- Type of health sector (public, private; hospital, community health services);
- Location of health facility (urban, rural);
- Management and leadership level (front-line/middle, senior).

## Discussion

Descriptive statistics will be used to summarise information on training and development programmes which are used to improve the competence of health management and leadership workforces and the acceptability of such programmes to participants. The review will identify common characteristics of: (1) interventions found to have improved the competence of participants, and (2) those interventions considered to be ineffective. Success of interventions or otherwise will be determined by examining outcomes and impact at the individual level only. If possible, the effects of such interventions on organisational performance, mediated through improved competence, will be identified.

The contextual effects of health settings on the interventions, ie, the relationship between health contexts and the interventions required to improve management and leadership competence, will be examined. This will help guide the applicability of the findings of the review to other health settings not identified by the included studies. If possible, any relationship between management levels and effectiveness of interventions will also be investigated. The outcome of this systematic review will be an understanding of the evidence for the relationship between training and professional development interventions and improved health management and leadership competence.

## Acknowledgments

The authors wish to thank Vanessa Jordan of the University of Auckland, Auckland, New Zealand for reviewing the methods section of the protocol.

## Ethical issues

Not applicable.

## Competing interests

Authors declare that they have no competing interests.

## Authors’ contributions

The protocol was conceived by ROA and NN. Both authors contributed to the development of the protocol. ROA wrote the draft copy and final version of the manuscript. NN, KAW, ZL, and AD commented on the draft copy and revised the final version of the manuscript. All authors approved the final version of the manuscript.

## Authors’ affiliations

^1^Health Systems Section, School of Population Health, University of Auckland, Auckland, New Zealand. ^2^Department of General Practice and Primary Health Care, School of Population Health, University of Auckland, Auckland, New Zealand. ^3^Department of Public Health, School of Psychology and Public Health, La Trobe University, Melbourne, Australia.

## Appendix 1

### Search Sources

The following electronic databases and catalogues will be searched:

- CENTRAL
- CINAHL
- Cochrane databases of Systematic Reviews (CDSR)
- Current contents
- Database of Abstracts of Review of Effects (DARE)
- Database of Public Health Effectiveness Reviews (DOPHER)
- EMBASE
- ERIC
- Health Promis (Database of the Health Development Agency)
- Health Management Information Consortium (HMIC)
- Health Services Technology Assessment Texts (HSTAT)
- JSTORE
- MEDLINE
- Public Affairs Information Service (PAIS)
- PsychINFO
- ScienceDirect
- Scopus
- System for Information on Grey Literature in Europe Archive (SIGLE)
- Trials Register of Public Health Interventions (TROPHI)
